# 

**Published:** 1911-07-01

**Authors:** 


### BUILDINGS CONTEMPLATED AND IN PROGRESS.

Axminster.—For the rebuilding of Axminster Cottage Hospital a site has been purchased and plans by a London architect have been accepted. It is hoped that the foundation-stone will be laid in August.

Bradford.—Mr. W. Williamson, the city architect, has designed the new Sanatorium buildings which are to be erected at Bradford.

Budapest.—It is stated that the "Hauptstädtische Finanz-Kommission," Budapest, is acquiring a site adjoining the Vácer Strasse on which to build a hospital with accommodation for 800 beds.

Dumfries.—The managers of the Dumfries Royal Infirmary contemplate additions estimated to cost about £10,000.

Exeter.—Additions are contemplated to the workhouse. Plans may be seen at the office of the architect, Mr. R. M. Challice, 14 Bedford Circus, Exeter.

Fulham.—Mr. A. Saxon Snell, F.R.I.B.A., is architect for the dispensary extension at Fulham Infirmary. Tenders are to be delivered by June 29.

Gateshead.—Messrs. Hine and Pegg, of London, are architects for the Lunatic Asylum, near Stannington and Morpeth, for which estimates are now invited.

Glasgow.—The Glasgow Parish Council contemplate the formation of an epileptic colony at Muckcroft at a cost of £43,520.

Goole.—Messrs. Ledgard and Son, of Leeds, are the builders accepted for the Goole Cottage Hospital buildings; the work is estimated to cost £5,500.

Hallwood (near Grenoside).—For the small-pox hospital an estimated cost of £2,000 is given. Particulars may be had from the Surveyor, Wortley (Yorks) Rural District Council.

Hinckley.—An inquiry has been held at the Council offices, Hinckley, into the application by the Urban and Rural Councils for a Joint Hospital. Mr. Walker is the consulting architect.

Hull.—The Hospital Extension Committee of the Board of Guardians have approved a design for the proposed new Hospital to be erected contiguous to the Workhouse. Accommodation is provided for 156 beds, with three isolation wards. The plans are being forwarded to the Local Government Board for approval.

Macclesfield.—Mr. Hartley Hacking, of Manchester, is architect for the alterations at the Macclesfield Infirmary. Tenders have been received and the lowest accepted, which amounts to £988. The highst of five is £1,125.

Portsmouth. — For the extension of Portsmouth Infirmary a tender of £39,989 by J. Crockerell, of Southsea, has been accepted. Mr. G. E. Smith, it will be remembered, is the architect.

Rochdale.—Mr. H. H. Clough, of Rochdale, is architect for the Nurses' Home to be erected at the Workhouse. Tenders have just been submitted.

South Hants.—A tender has been accepted, owing to the munificence of Miss Burrell, of Botley, for the erection of an out-patients' wing at the Royal South Hants and Southampton Hospital. The cost of the works, including lighting and professional fees, is estimated at £10,609. See The Hospital of December 10, 1910.

Tewkesbury.—An inquiry has been held at Tewkesbury by the Local Government Board regarding a loan of £5,000 for the purpose of extending the Tredington Hospital. Mr. James Villar, architect, gave evidence explaining the arrangements, which include separate blocks for scarlet fever, diphtheria, and small-pox, but it was suggested that the last named should occupy a different site.

Thames Ditton.—A new ward has been opened at Thames Ditton Cottage Hospital. The management are indebted to Mr. A. J. Style, F.R.I.B.A., the honorary treasurer, for his generous services as architect.

Woodford.—A new ward and other additions have been opened at the Woodford Jubilee Hospital. The cost of the work has amounted to £1,029, leaving a balance to the endowment fund of over £50.

Woodhouse Eaves.—Messrs. Searle and Riley are architects for the Women's Convalescent Home now being erected at Woodhouse Eaves, Charnwood Forest, for the Leicester and County Saturday Hospital Society. An account of the opening appears in another column.

### VACCINATION FEES.

The question of fees paid to public vaccinators in the district of Bermondsey has again been raised in the House of Commons. Mr. Joynson-Hicks asked the President of the Local Government Board whether, seeing that the proper charge for public vaccination is 4s. 2d. at the surgery and 6s. 8d. at the patient's house, he has sanctioned Dr. Jaques charging 6s. 8d. for each child and adult he vaccinates at a place other than the patient's home. In reply, Mr. Burns stated that Dr. Jaques had been appointed by the Board as an authorised teacher of vaccination. In this capacity he receives a fee of 5s. in respect of every successful primary vaccination of any child, which may be performed elsewhere than at the home of the child vaccinated, but at a place approved by the Board. Dr. Jaques is only concerned with the vaccination of adults in his capacity of public vaccinator, and the fee in respect of such cases is 2s. 6d. when the operation is performed elsewhere than at the home of the patient. In addition the public vaccinator receives a birth fee of 1s. 8d. in respect of each child born within his district.

The Leicester Infirmary has received £100 under the will of the late Mr. S. S. M. Howkins, and £50 under the will of Mrs. M. A. Hodgson, and £200 from the late Mr. F. R. Morley, who has also bequeathed £50 to the Leicester Children's Hospital.

The report for the year 1910 of the medical officer of health for the Metropolitan Borough of Hampstead shows that during the year the birth, marriage, and death rates were respectively 14.0, 13.2, and 8.6 per 1,000 living population as compared with rates of 13.9, 14.4, and 8.9 respectively for the year previous. The infantile death rate was 60 per thousand births as against 74 for the previous year. This rate is the lowest ever recorded for the borough. Erysipelas yielded 32 cases with one death, the attack rate being 0.33 per 1,000. Two cases of epidemic cerebro-spinal meningitis were reported, both of which died. Post-mortem examination showed that a mistake had been made in diagnosis. Tuberculosis caused 80 deaths and a death-rate of 0.83.

### THE QUESTION BOX.

### WHAT HOT-WATER BOTTLES LAST?

Sir,—At the Royal Liverpool County Hospital for Children at Heswall we are experiencing great difficulty in finding suitable hot-water bottles. We have open air wards of which the flooring is asphalt. In the cold weather each child requires one hot bottle, sometimes three. They frequently kick them out of bed, or the bottles sometimes are dropped. Of course stone ones break at once on the floor. We have tried copper ones corrugated, and one of steel pressed out, but that was attacked by rust. Can any of your readers give us any assistance, as so far we have not found any bottle to last?—Yours truly,

E. J. Deane, Hon. Secretary.

14 Dale Street, Liverpool.

### GIFTS.

Mrs. Eliza Rolls, of Arundel, has bequeathed £500 to Worthing Hospital.

Mrs. Emma Esther Deacon, of Kensington, has bequeathed £300 to the Royal Hospital for Incurables.

The Speaker of the House of Commons is one of the executors of the will of the late Miss Charlotte Antonia Sulivan, a niece of Lord Palmerston, who has bequeathed the Parsons Green Club, the Old Hope Coffee Tavern, and £2,000 to the Charity Commissioners. There is also a bequest to the West London Hospital.

### THE WEEK'S NOVELTIES.

Messrs. J. G. Graves, of Sheffield, have left the ill-dressed institutional man little excuse. For 5s. down and six further payments of 5s., extending over six months, they supply a suit, of which, as Messrs. Graves themselves point out, you and you alone are to judge the value. Any readers who fancy that good clothes and high prices are inseparable should drop a card for samples of Messrs. Graves' patterns.

### COMING EVENTS OF THE WEEK.

[Communications for this column must be sent to 29 Southampton Street, Strand, before noon on Wednesday.]

Tuesday, July 4.—National Hospital, Queen's Square, W.C. Lecture, "Paraplegia," by Dr. A. Turner, 3.30 p.m.
Guy's Hospital, St. Thomas's Street, S.E. Annual Garden Party.

Wednesday, July 5.—Central London Ophthalmic Hospital, Judd Street (near St. Pancras station), W.C. Duchess of Albany lays foundation stone of new buildings, 3 p.m.
London Homœopathic Hospital, Great Ormond Street, W.C. Princess Louise (Duchess of Argyll) opens the Sir Henry Tyler extension wing, 4 p.m.
Royal Hospital for Incurables, Putney Heath. Garden Party.

Thursday, July 6.—Salford Royal Hospital. Official opening of the King Edward Memorial wing, new wards, and Nurses' Home by Lord Shuttleworth, at 3 p.m. Sir W. Mather will preside.

Friday, July 7.—National Hospital, Queen's Square, W.C. Lecture, "Cerebellar Disease," by Dr. G. Holmes, 3.30 p.m.
West London Hospital, Hammersmith Road. Lecture, "Clinical Pathology," by Dr. Bernstein, 5 p.m.

### MEDICAL APPOINTMENTS.

The following appointments have been made at Leicester Infirmary: Senior House Surgeon, Mr. Cuthbert Adeney, M.B., B.S., F.R.C.S., late house physician St. Thomas's Hospital, and casualty house surgeon and house surgeon to the Hull Royal Infirmary. Second House Surgeon, Mr. M. M. Morrison, M.B., Ch.B. Ed., assistant house surgeon to the Leicester Infirmary. Assistant House Physician, Mr. Robert Findley, M.B., Ch.B. Glas.

### APPOINTMENTS VACANT.

Full particulars of these vacancies can be obtained on reference to our advertisement columns:

Addenbrooke's Hospital, Cambridge.—House Physician and Second House Surgeon.

Coventry and Warwickshire Hospital.—Junior House Surgeon.

Hospital for Sick Children, Great Ormond Street, W.C.—House Physician and House Surgeon.

Leicester Infirmary.—Assistant House Surgeon.

Manchester Children's Hospital.—Assistant Medical Officer.

Royal Albert Hospital, Devonport.—Assistant House Surgeon.

Sheffield Royal Infirmary.—Seventh Resident Medical Officer.

Somerset and Bath Asylum.—Assistant Medical Officer.

